# A Quantitative Analysis of Complexity of Human Pathogen-Specific CD4 T Cell Responses in Healthy *M*. *tuberculosis* Infected South Africans

**DOI:** 10.1371/journal.ppat.1005760

**Published:** 2016-07-13

**Authors:** Cecilia S. Lindestam Arlehamn, Denise M. McKinney, Chelsea Carpenter, Sinu Paul, Virginie Rozot, Edward Makgotlho, Yolande Gregg, Michele van Rooyen, Joel D. Ernst, Mark Hatherill, Willem A. Hanekom, Bjoern Peters, Thomas J. Scriba, Alessandro Sette

**Affiliations:** 1 La Jolla Institute for Allergy and Immunology, Department of Vaccine Discovery, La Jolla, California, United States of America; 2 South African Tuberculosis Vaccine Initiative, Institute of Infectious Disease and Molecular Medicine, and Division of Immunology, Department of Pathology, University of Cape Town, Cape Town, South Africa; 3 Department of Medicine, Division of Infectious Diseases, New York University School of Medicine, New York, New York, United States of America; New Jersey Medical School, UNITED STATES

## Abstract

We performed a quantitative analysis of the HLA restriction, antigen and epitope specificity of human pathogen specific responses in healthy individuals infected with *M*. *tuberculosis* (Mtb), in a South African cohort as a test case. The results estimate the breadth of T cell responses for the first time in the context of an infection and human population setting. We determined the epitope repertoire of eleven representative Mtb antigens and a large panel of previously defined Mtb epitopes. We estimated that our analytic methods detected 50–75% of the total response in a cohort of 63 individuals. As expected, responses were highly heterogeneous, with responses to a total of 125 epitopes detected. The 66 top epitopes provided 80% coverage of the responses identified in our study. Using a panel of 48 HLA class II-transfected antigen-presenting cells, we determined HLA class II restrictions for 278 epitope/donor recognition events (36% of the total). The majority of epitopes were restricted by multiple HLA alleles, and 380 different epitope/HLA combinations comprised less than 30% of the estimated Mtb-specific response. Our results underline the complexity of human T cell responses at a population level. Efforts to capture and characterize this broad and highly HLA promiscuous Mtb-specific T cell epitope repertoire will require significant peptide multiplexing efforts. We show that a comprehensive “megapool” of Mtb peptides captured a large fraction of the Mtb-specific T cells and can be used to characterize this response.

## Author Summary

Human pathogen-specific immune responses are tremendously complex and the techniques to study them ever expanding. There is an urgent need for a quantitative analysis and better understanding of pathogen-specific immune responses. *Mycobacterium tuberculosis* (Mtb) is one of the leading causes of mortality due to an infectious agent worldwide. Here, we were able to quantify the Mtb-specific response in healthy individuals with Mtb infection from South Africa. The response is highly diverse and 66 epitopes are required to capture 80% of the total reactivity. Our study also show that the majority of the identified epitopes are restricted by multiple HLA alleles. Thus, technical advances are required to capture and characterize the complete pathogen-specific response. This study demonstrates further that the approach combining identified epitopes into “megapools” allows capturing a large fraction of the total reactivity. This suggests that this technique is generally applicable to the characterization of immunity to other complex pathogens. Together, our data provide for the first time a quantitative analysis of the complex pathogen-specific T cell response and provide a new understanding of human infections in a natural infection setting.

## Introduction

Antigen-specific CD4 T cell responses are functionally very diverse, and have been classified into several different Th subsets based on their expression of distinct chemokine receptors, secretion of effector cytokines, and different transcriptional programs and differentiation states [1,2]. The depth to which these responses can be characterized has increased dramatically in recent years. Novel technologies, such as multiparameter flow cytometry, cytometry by time of flight (CyTOF), and single-cell transcriptomic profiling, which allow simultaneous characterization of many functional and phenotypic markers are revealing an unprecedented degree of complexity in immune responses [3–8].

Human antigen-specific CD4 T cell responses are also highly complex at the level of HLA restriction, antigen and epitope specificity [9–12]. Humans express HLA class II α/β heterodimers encoded by four different β-chain loci, DRB1, DRB3/4/5, DQB1 and DPB1, as well as corresponding α-chain loci DRA1, DQA1 and DPA1 [13]. All loci, with the exception of the DR α-chain, are extremely polymorphic and more than 1,500 alleles have been identified to date [14]. As a result, most individuals are heterozygous at these loci and express up to eight different HLA class II molecules.

At the antigen and epitope levels, especially in complex organisms, it is clear that T cell responses are also highly complex, often involving tens of different antigens and hundreds of epitopes [10–12,15,16]. Patterns of immunodominance in humans are much less narrow than those observed in murine, genetically homogenous model systems. While mechanisms of immunodominance and breadth of T cell responses have been comprehensively analyzed in murine systems and to some degree in humans [17–20], a quantitative assessment of the complexity of responses at the population level, in the course of natural infections, is lacking. Most immuno-profiling studies have thus targeted individual antigens or a limited set of epitopes under the assumption that these represent the entire pathogen-specific response. It is currently unknown to what degree underestimating the true complexity might impact the outcomes generated by immuno-profiling studies.

Tuberculosis (TB) is the leading cause of mortality, alongside HIV, in South Africa and worldwide due to a single infectious agent [21]. South Africa has the highest rate of incident TB in the world with almost 1 in every 100 persons developing active TB each year [21] and an estimated 70–80% of the adult population has latent Mtb infection [22].

Several cytokines are involved in T cell responses against Mtb. Individuals with genetic defects in the IL-12 pathway or the IFNγ-receptor have increased susceptibility to mycobacteria [23–25], providing evidence that IFNγ is necessary for protective immunity against Mtb. Indeed, CD4 T cell responses to Mtb are contained in a CXCR3^+^CCR6^+^ Th subset, cells that produce IFNγ, IL-2 and TNFα [10], and a mutation leading to the loss of the CXCR3^+^CCR6^+^ lineage specific transcription factor RORC leads to mycobacteriosis [26]. Increased production of Th2 cytokines have been reported in patients with active TB compared to healthy controls with latent Mtb infection [27,28], while Mtb-specific CD4 T cells have also been shown to express IL-17 in response to PPD, Mtb lysate and more limited studies of defined peptide pools [29–32]. IL-10 has been shown to mediate a decreased ability to clear Mtb infection and the levels of IL-10 is increased in the serum of active TB patients [33,34].

Our previous work in a population of healthy Mtb-infected individuals from USA has shown that Mtb epitope-specific CD4 T cells are also very heterogeneous at a cohort level [10]. The breadth of responses was remarkably wide, with an average individual simultaneously recognizing 24 distinct epitopes. However, no antigen was recognized by all individuals, and 82 antigens were required to cover 80% of the T cell response.

Here we undertook a comprehensive analysis of the epitope specificity and HLA restriction of human T cell responses associated with Mtb-infection in a healthy cohort of 63 South African individuals from a setting endemic for TB. The results provide evidence of striking heterogeneity in recognition of different Mtb antigens and overall breadth of epitope responses. We identified a set of epitopes that provides good coverage of the Mtb peptide-specific T cell response restricted by an extensive variety of HLA class II molecules in an independent validation cohort.

## Results

### Mtb-specific T cell responses are polarized towards IFNγ expression

A total of 63 HIV-negative adults were enrolled into our screening cohort. All, except one, were QFT positive. The QFT-negative donor was previously QFT positive and was therefore a QFT reverter, who may still be infected with Mtb. Demographic characteristics are reported in Table 1. Traditional approaches to T cell epitope discovery typically rely on methods that detect IFNγ expression. To determine if this Th1-biased approach led to a bias in epitope discovery, we compared identification of T cell responses to peptides by measuring IL-5 (Th2 cytokine), IL-17 (Th17 cells), IL-10 (regulatory cytokine) in addition to IFNγ (Th1 cytokine) in our ex vivo ELISPOT assays. We anticipated the vast majority of responses to be mediated by CD4 T cells since earlier studies using the exact same methodology found that more than 95% of responses were HLA class II restricted [10,35]. We limited these experiments to peptides spanning a set of 11 proteins contained in TB vaccines currently in clinical trials or contained in IGRAs (termed “TB Vaccine and IGRA antigens”; Table 2).

**Table 1:** Demographic characteristics of enrolled participants.

Cohort	Screening	Validation	Negative control
Total participants enrolled, n	63	60	17
Male, n (%)	41 (65%)	32 (53%)	8 (47%)
Median age, years (range)	23 (20–26)	17 (14–20)	18 (17–19)
Ethnicity, n (%)			
Colored (mixed race)	58 (92%)	45 (75%)	13 (76%)
Black African	3 (5%)	15 (25%)	4 (24%)
Caucasian	2 (3%)	0 (0%)	0 (0%)
HIV seropositive, n	0	Not tested	Not tested
Quantiferon Gold In-Tube positive, n (%)	62 (98%)	60 (100%)	0 (0%)
Tuberculin Skin Test	Not tested	38 (97%) / 39	8 (53%) / 15
n positive (%) / n tested			

**Table 2:** List of TB Vaccine and IGRA antigens screened with overlapping peptides.

Rv No.	Synonym(s)^a^	Category	Vaccine candidate	Protein length (aa)	No. peptides^b^
Rv0125	Mtb32A, pepA	Intermediary metabolism and respiration	M72	355	69
Rv0288	EsxH, TB10.4, CFP7	Cell wall and cell processes	Aeras402, HyVac4	96	18
Rv1196	Mtb39A, PPE18	PE/PPE	M72	391	77
Rv1813c	-	Conserved hypotheticals	ID93	143	27
Rv1886c	Ag85B	Lipid metabolism	Aeras402, H1, HyVac4	325	63
Rv2608	PPE42	PE/PPE	ID93	580	114
Rv2660c	-	Conserved hypotheticals	H56	75	13
Rv3619c	EsxV, Mtb9.9D	Cell wall and cell processes	ID93	94	17
Rv3620c	EsxW	Cell wall and cell processes	ID93	98	18
Rv3804c	Ag85A	Lipid metabolism	MVA85A, Aeras402, Ad85A	338	66
Rv3874	CFP10	Cell wall and cell processes	-	100	18
Rv3875	ESAT-6	Cell wall and cell processes	H1, H56	95	17

^a^As indicated in Tuberculist [36].

^b^Number of 15-mer peptides overlapping by 10 amino acids (aa) screened per antigen.

In 12 participants, IFNγ responses accounted for nearly 96% of the recognition events, and for nearly 99% of the total response magnitude (Table 3). Cells expressing IL-5 or IL-10 were extremely rare and no IL-17-expressing cells were detected, demonstrating that responses to Mtb protein antigens were highly polarized towards IFNγ-producing T cell subsets. For all samples cell viability was above 90% and all cytokines were readily detectable in positive controls, demonstrating that the IFNγ polarization was not a technical artifact. Not a single instance of a donor/peptide pool combination that was positive for IL-5, IL-17 or IL-10, but negative for IFNγ, was observed. Thus, screening for IFNγ production correctly identified all positive donor/pool responses. Based on these results, all subsequent experiments and assays on remaining donors were performed with IFNγ ELISPOT assays.

**Table 3:** Responses to TB Vaccine and IGRA antigens are highly IFNγ polarized.

Cytokine	% positive pools^a^	% magnitude^b^
IFNγ	95.6	98.9
IL-5	1.6	0.4
IL-10	2.4	0.7
IL-17	0	0

^a^Percentage of positive pools out of the total number positive pools detected for all donors.

^b^Percentage of total magnitude of positive responses (SFC/10^6^) detected for all donors.

### Hierarchy in reactivity against TB Vaccine and IGRA antigens

The overall goal of our experiments was to broadly evaluate T cell responses in terms of epitope reactivity, immunogenicity, immunodominance, and HLA allele restriction in LTBI donors from the Western Cape region of South Africa, a setting where TB is endemic.

We tested all 63 donors for IFNγ reactivity to pools of overlapping peptides spanning the TB Vaccine and IGRA antigens. Positive pools were deconvoluted to identify individual T cell epitopes. Overall, 86% of donors recognized epitopes from at least one antigen, and on average 2 (range 0–8) different antigens per donor were recognized. This is consistent with our previous report that highlights the considerable breadth and inter-individual variability of epitope-specific responses in persons with LTBI [10].

**Fig 1:**
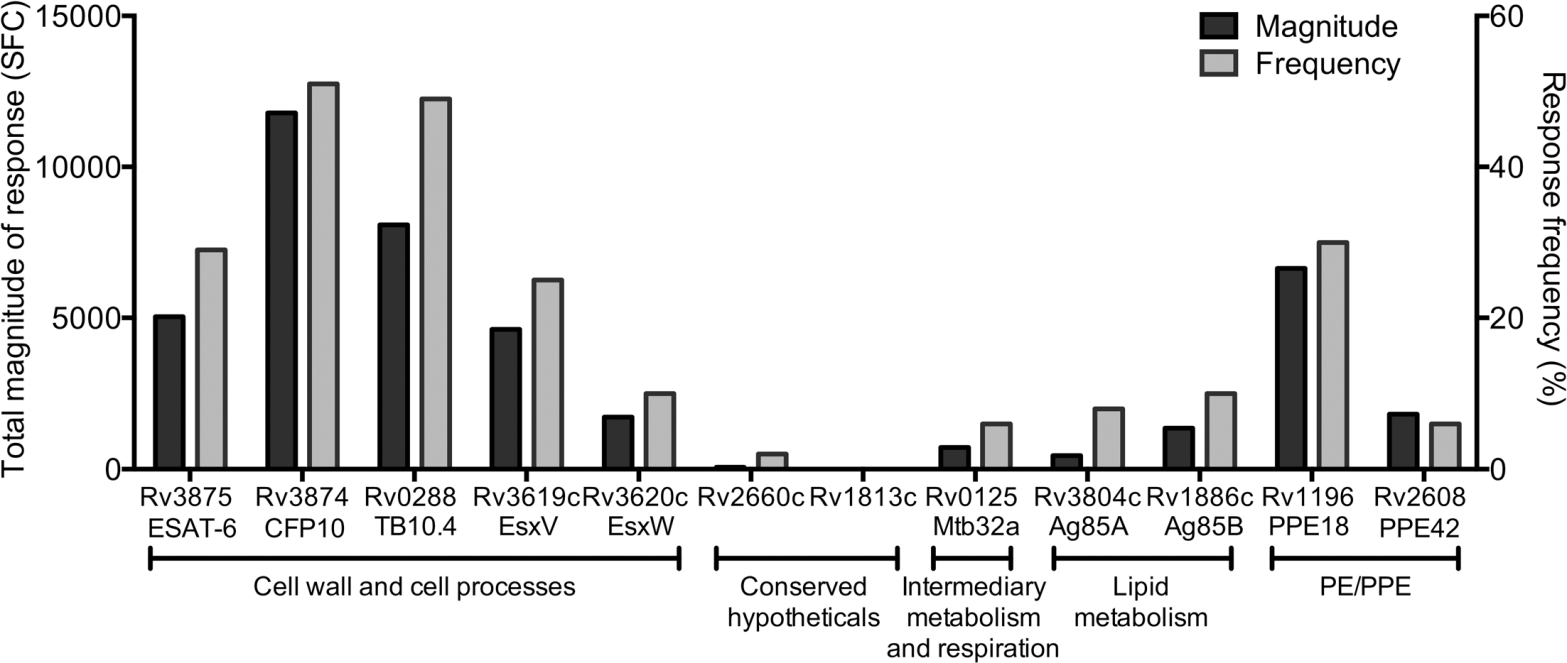
Hierarchy in T cell reactivity against TB Vaccine and IGRA antigens. Magnitude of responses, expressed as the total magnitude of response (black bars, left y-axis) or frequency of donors responding (grey bars, right y-axis), amongst the 63 donors. Rv number and synonyms for each antigen are indicated on the x-axis. Antigens were divided into protein categories as defined by Tuberculist [36]. All five antigens that are part of the cell wall and cell processes category are involved in the type VII secretion system [37].

The *ex vivo* reactivity, expressed in terms of total magnitude of response and response frequency to the different TB Vaccine and IGRA antigens, is shown in Fig 1. The most frequently recognized proteins were Rv3874 and Rv0288, which were recognized by more than 40% of the donors. Rv1196, Rv3875, and Rv3619c were recognized by more than 20% of the donors, while Rv3620c, Rv2660c, Rv0125, Rv3804c, Rv1886c, and Rv2608 were recognized in less than 10% of donors. No reactivity was observed to Rv1813c in this cohort. The same hierarchy of reactivity to these antigens was seen when considering the total magnitude of response (Fig 1).

Antigens classified according to the Tuberculist database [36], into the cell wall and cell processes category were the most immunogenic, followed by the PE/PPE category (Fig 1). Lower immunogenicity was detected for proteins classified as conserved hypotheticals, involved in intermediary metabolism and respiration, as well as lipid metabolism proteins.

### Identification of antigenic regions within TB Vaccine and IGRA antigens

**Fig 2:**
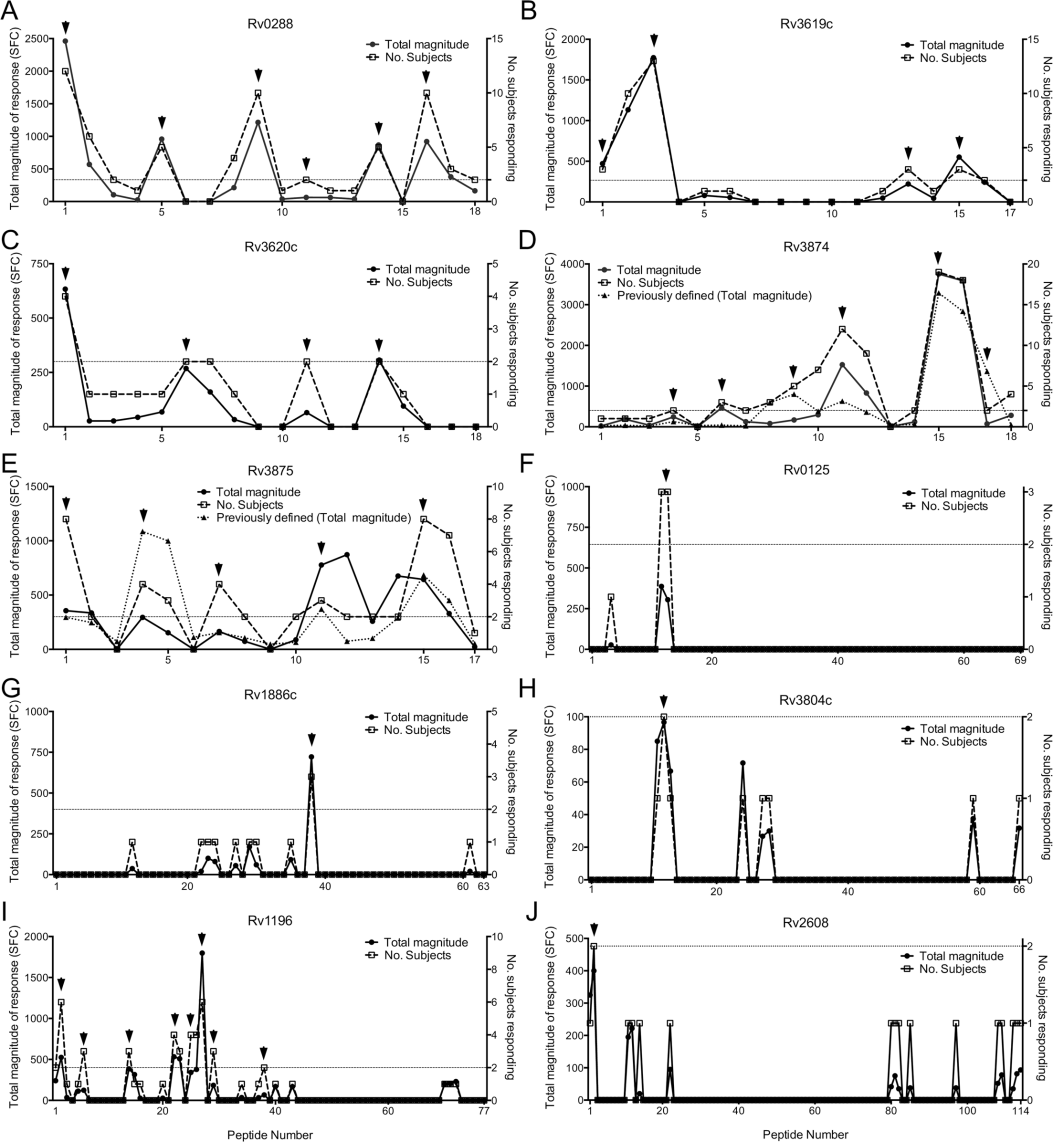
Identification of antigenic regions within the TB Vaccine and IGRA antigens. Magnitude of responses, expressed as the total magnitude of response (black dots, solid line, left y-axis) or number of responding donors (squares, dashed line, right y-axis) identified for Rv0288 (A), Rv3619c (B), Rv3620c (C), Rv3874 (D), Rv3875 (E), Rv0125 (F), Rv1886 (G), Rv3804c (H), Rv1196 (I), and Rv2608 (J). X-axes indicate peptide number of overlapping peptides spanning the entire protein. Dashed horizontal lines indicates 2 responding donors, and arrows indicate non-redundant antigenic regions. Cell wall and cell processes (A-E), Intermediary metabolism and respiration (F), Lipid metabolism (G, H) and PE/PPE antigens (I, J). Previously defined antigenic regions [35] (also expressed as total magnitude of response) are indicated by black triangles and dotted lines (left y-axis) for Rv3874 (D) and Rv3875 (E).

Next, we undertook a more detailed analysis to identify individual reactive peptides within the TB Vaccine and IGRA antigens. A total of 64 peptides were recognized by two or more donors. When responses to two consecutive overlapping peptides were positive, the peptide with the highest magnitude and response frequency was chosen as the optimal epitope. We have previously shown that responses to two consecutive overlapping peptides typically map to the same single epitope [35]. Using these criteria, the recognition of 64 peptides defined 37 distinct antigenic regions, (Fig 2 and S1 Table). In the case of Rv0288, 6 antigenic regions were defined, and for Rv3619c and Rv3620c 4 regions each (Fig 2A–2C). For Rv3874 and Rv3875, 6 and 5 regions were defined, respectively (Fig 2D and 2E). Interestingly these antigenic regions were almost identical to those previously defined in the cohort of IGRA-positive donors from San Diego [35,38]. We only identified one antigenic region each in Rv0125, Rv1886c, Rv3804c, or Rv2608 (Fig 2F–2I), while 8 antigenic regions were defined in Rv1196 (Fig 2J). Due to their low recognition frequency, no clear antigenic regions were detected in the case of Rv2660c or Rv1813c. The number of responding donors corresponded well to the total magnitude of response within the cohort. When we analyzed peptides that were recognized by a high frequency of responders, none were dominated by a single responding donor (Fig 2).

### Towards “total” TB epitope reactivity and comprehensive response coverage

We also assembled a set of peptides previously described in studies of ex vivo human CD4 T cell epitope responses to Mtb [10,35,39–41]. A total of 253 epitopes, derived from 94 Mtb antigens, were screened for recognition by IFNγ ELISPOT assay in the 63 South African adult donors. These experiments defined 38 non-redundant epitopes recognized by 2 or more donors in the cohort (S2 Table). Nine epitopes identified amongst the TB Vaccine and IGRA antigens were also found to be reactive in the previously described epitope set. These epitopes were highly immunodominant and accounted for 50% and 54% of the T cell reactivity detected amongst the TB Vaccine and IGRA antigens and previously described epitope set, respectively. This result is consistent with previous estimates that bioinformatic prediction for promiscuous epitopes captures about 50% of the total reactivity [10,42].

In conclusion, a set of 66 non-redundant epitopes derived from TB Vaccine and IGRA antigens and previously described epitopes provide comprehensive coverage of the donor cohort investigated.

### Overall breadth and multispecificity of responses

**Fig 3:**
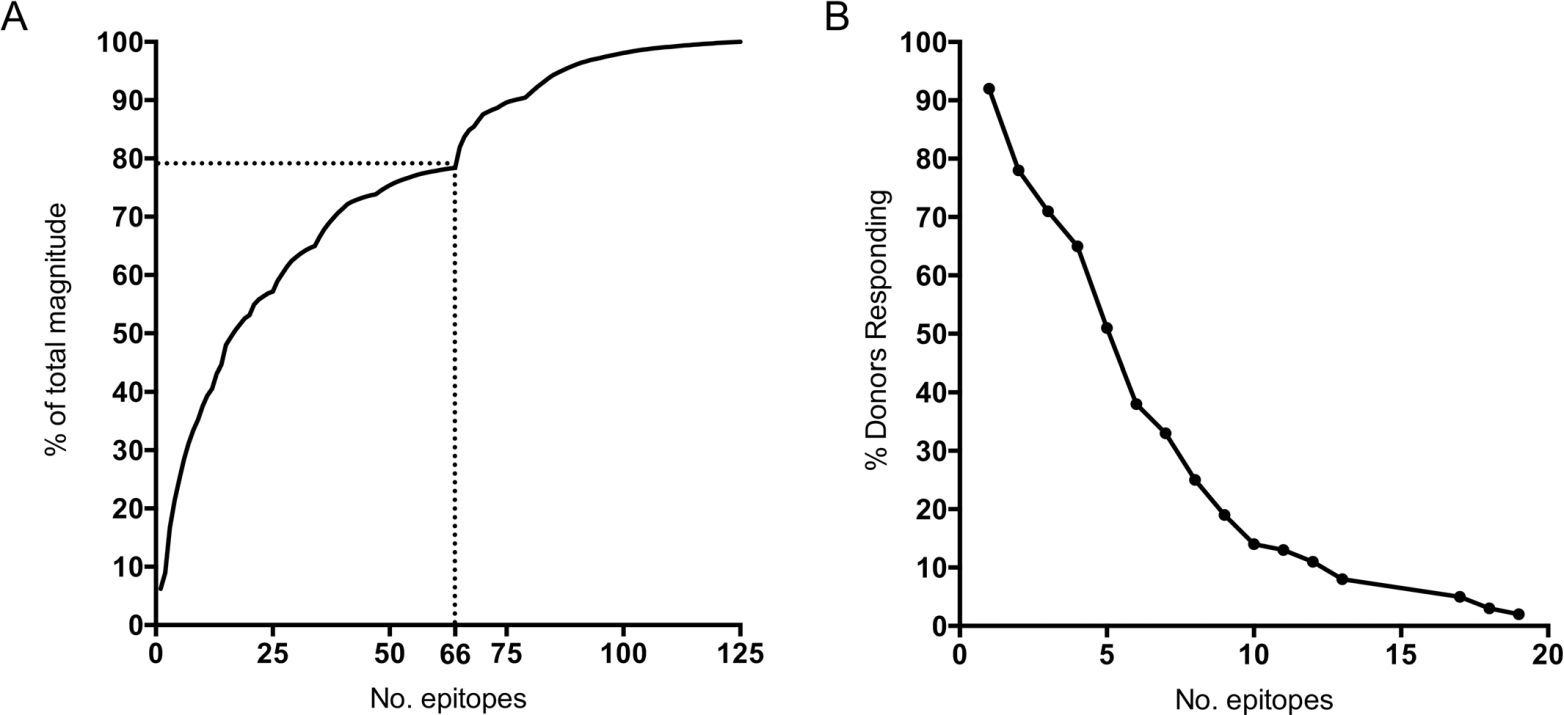
Breadth and dominance of T cell epitopes in Mtb antigens. (A) All epitopes (n = 125) as a percentage of the total magnitude of response ranked on the basis of magnitude of T cell response and the 66 epitopes identified from TB vaccine and IGRA antigens and previously described epitopes. The dotted line indicates the 66 most immunodominant epitopes. (B) Proportion of the 63 donors who respond to the indicated number of epitopes of the top 66 identified epitopes.

We previously showed in a IGRA-positive donor cohort from the San Diego region that responses were highly heterogeneous [10]. Here we investigated whether this is also true for the present study. A total of 235 peptides were recognized in at least one donor. After removal of redundant epitopes, 125 unique epitopes were recognized by at least 1 donor and the 66 epitopes identified from TB Vaccine and IGRA antigens and previously described epitopes captured 80% of the response (Fig 3A). To investigate whether the IGRA antigens accounted for the majority of the reactivity we divided the 125 unique epitopes into whether they mapped to IGRA or to non-IGRA antigens. Significantly higher reactivity was observed to the other (non-IGRA) antigens (S1A Fig). Furthermore, we also stratified the 125 peptides according to whether they were co-expressed by Mtb and BCG or known to be absent in BCG (according to Behr et al. [43]). Significantly higher reactivity was observed to the antigens shared by Mtb/BCG than to the antigens only expressed by Mtb (S1B Fig). Each donor recognized an average of 5 of the 66 epitopes (range 0 to 19). We further found that 92% of donors recognized at least one epitope and 70% of donors recognized at least three epitopes (Fig 3B). To enable investigation of responses to these epitopes in cohorts where less PBMC numbers were available we pooled the 125 unique epitopes as one peptide pool and the top 66 epitopes as another, see below.

### Determination of HLA restriction of Mtb epitopes

To further understand the degree of complexity of the responses in the donor population, we sought to determine the restricting class II HLA molecule(s) of each epitope. HLA restrictions can be determined by testing for PBMC responses to each epitope recognized by that donor when the peptide is presented by single-HLA-molecule transfected antigen presenting cell lines that match the HLA type of the donor [44]. Genotypes of each individual donor at the HLA class II DR, DP and DQ loci was determined [45]. A recently described comprehensive panel of 48 HLA transfected cell lines [44] allowed coverage of 63% of the identified HLA class II alleles in the donor cohort (S3 Table).

A total of 778 different positive donor/epitope combinations corresponded to 5,623 possible HLA/epitope/donor combinations, since each donor expresses up to two alleles at each of the four HLA class II loci. The panel of transfected cell lines allowed HLA restriction to be determined for 3,739 (66%) of these combinations. Because PBMCs from the South African donors were limited, not all possible HLA restrictions for each epitope could be tested. In those instances, we focused on determining the restriction of the epitopes associated with the highest response magnitudes.

**Fig 4:**
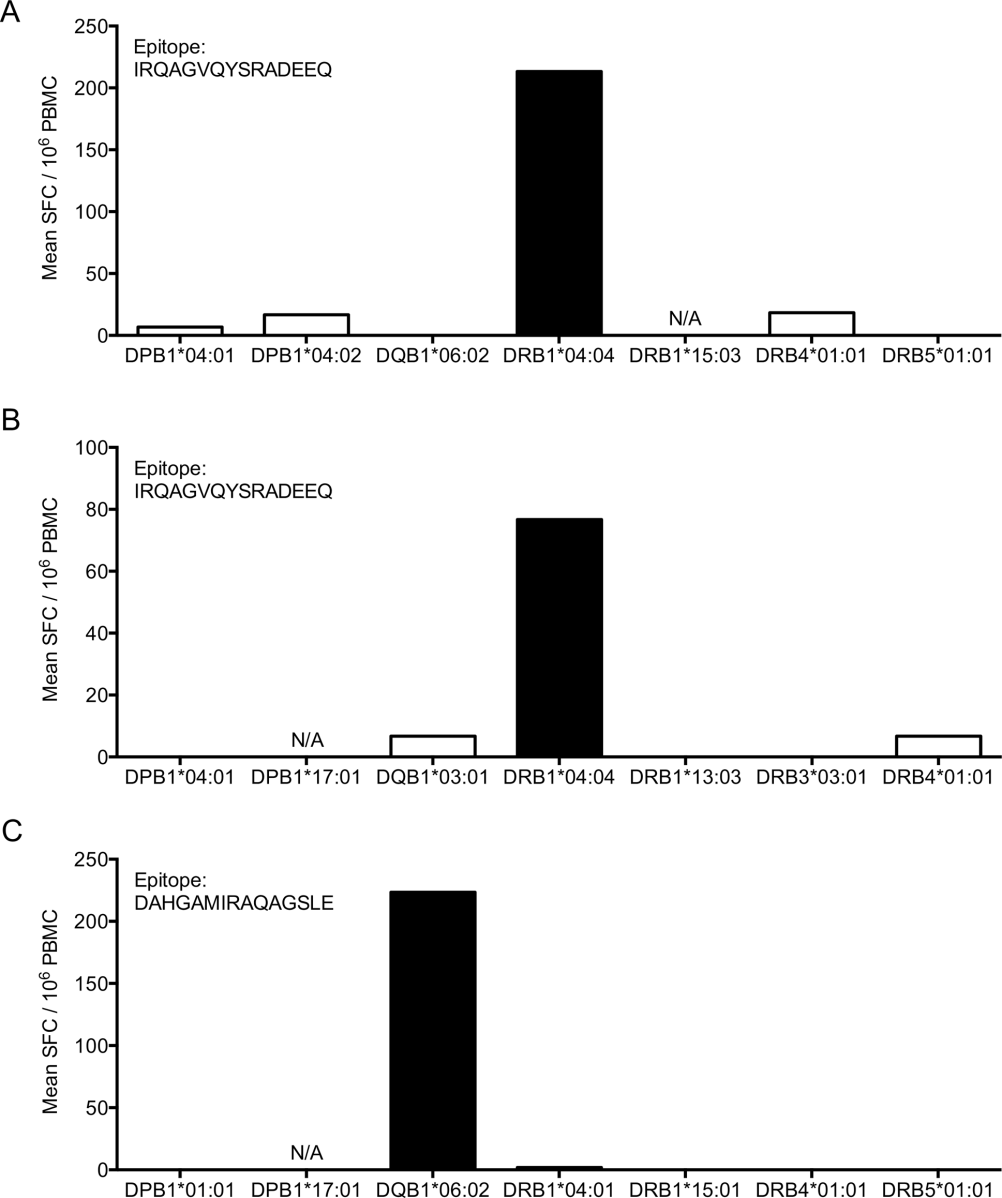
Determination of HLA restriction of Mtb epitopes using HLA-transfected cell lines. Representative examples showing determination of HLA restriction for two epitopes in three donors. (A-C) PBMCs were incubated with peptide-pulsed cell lines transfected with each individual HLA molecule that matched the HLA alleles of the PBMC donor. IFNγ release was measured by ELISPOT. Positive responses (black bars, p<0.05), negative responses (white bars). N/A indicates cell line not available for the HLA allele.

Overall, we were able to define a total of 2,916 HLA allele/epitope restrictions, corresponding to 52% of the total and 78% of the epitope/donor combinations for which HLA transfected cell lines were available. Representative data is shown for three epitope/donor combinations in Fig 4, where a response was detected when the peptide was presented by DRB1*04:04 (Fig 4A and 4B) or DQB1*06:02 (Fig 4C) transfected cells, but not by any of the other lines transfected with HLA molecules expressed by the donor.

### Promiscuity and penetrance of HLA restriction at the population level

**Fig 5:**
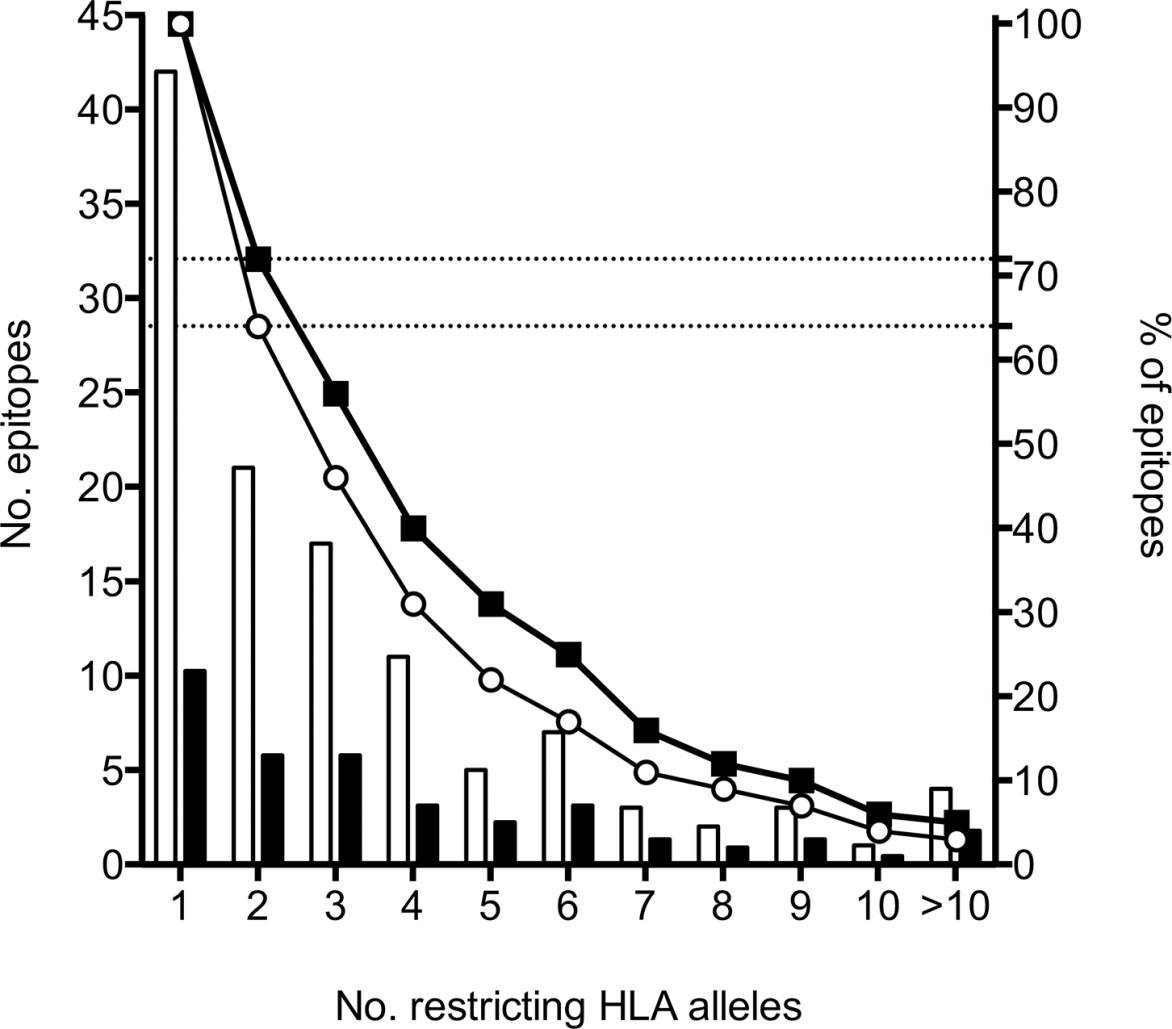
Promiscuity of HLA restrictions. Number of epitopes identified (left y-axis) and the corresponding number of restricting HLA alleles per epitope (x-axis). Percentage of epitopes (right y-axis) restricted by ≥ indicated number of HLA alleles. All restrictions identified (open bars and circles). Restrictions in epitopes tested in ≥2 subjects (black bars and squares).

Experimentally testing 2,916 HLA/epitope/donor combinations allowed us to determine 519 HLA/epitope/donor restrictions (S4 Table). A summary of 37 restrictions identified in 3 or more subjects, corresponding to 19 unique epitopes, is presented in Table 4. Given the promiscuous nature of HLA class II-restricted responses, we also determined to what extent the recognized epitopes were associated with promiscuous recognition (i.e. restricted by more than one HLA molecule encoded by different HLA loci, or within the same locus but differing sufficiently—by more than the 2-digit level—to be classified as distinct HLA subtypes) [46]. Sixty-four % of all epitopes and 72% of epitopes tested in 2 or more donors were associated with promiscuous recognition (Fig 5).

**Table 4:** HLA restriction and penetrance of dominant epitopes.

Antigen	Epitope sequence	Restricting HLA allele	Donors with allele-restricted response, n	Donors with HLA allele tested, n	Penetrance (%)
Rv3874	AQAAVVRFQEAANKQ	DRB5*01:01	6	7	86%
Rv3874	QAAVVRFQEAANKQK	DRB5*01:01	6	10	60%
Rv3619c	DAHGAMIRAQAGSLE	DQB1*06:02	5	5	100%
Rv3874	ISTNIRQAGVQYSRA	DRB3*02:02	5	7	71%
Rv3874	EISTNIRQAGVQYSR	DRB3*02:02	5	8	63%
Rv3874	EISTNIRQAGVQYSR	DRB1*04:01	5	9	56%
Rv3874	QAAVVRFQEAANKQK	DQB1*06:02	5	9	56%
Rv3874	ISTNIRQAGVQYSRA	DQB1*06:02	4	4	100%
Rv1195	MHVSFVMAYPEMLAA	DQB1*06:02	4	4	100%
Rv2031c	AYGSFVRTVSLPVGA	DRB1*07:01	4	5	80%
Rv3874	EISTNIRQAGVQYSR	DRB1*04:04	4	5	80%
Rv3874	ISTNIRQAGVQYSRA	DRB1*04:04	4	5	80%
Rv3874	VRFQEAANKQKQELD	DRB5*01:01	4	6	67%
Rv1196	ALPPEINSARMYAGP	DQB1*06:02	3	3	100%
Rv1788^**a**^	MSFVTTQPEALAAAA	DRB1*07:01	3	3	100%
Rv3874	IRQAGVQYSRADEEQ	DRB1*04:04	3	4	75%
Rv3136	LLGQNTAAIAAIEAQ	DRB3*02:02	3	4	75%
Rv1195	MHVSFVMAYPEMLAA	DRB5*01:01	3	4	75%
Rv0288^**a**^	MSQIMYNYPAMMAHA	DRB1*15:01	3	4	75%
Rv3619c	QFGDVDAHGAMIRAQ	DQB1*06:02	3	4	75%
Rv3615c	VDLAKSLRIAAKIYS	DRB3*03:01	3	4	75%
Rv3874	AAVVRFQEAANKQKQ	DRB5*01:01	3	5	60%
Rv3874	EISTNIRQAGVQYSR	DQB1*06:02	3	5	60%
Rv3874	EISTNIRQAGVQYSR	DPB1*01:01	3	5	60%
Rv1196	TPAIAVNEAEYGEMW	DRB3*02:02	3	5	60%
Rv0287^**a**^	AAFQAAHARFVAAAA	DRB1*07:01	3	6	50%
Rv0280^**a**^	GINTIPIAINEAEYV	DRB3*02:02	3	6	50%
Rv3874	ISTNIRQAGVQYSRA	DPB1*01:01	3	6	50%
Rv3874	QAAVVRFQEAANKQK	DRB3*02:02	3	6	50%
Rv3615c	VDLAKSLRIAAKIYS	DRB1*11:01	3	6	50%
Rv3615c	VDLAKSLRIAAKIYS	DQB1*06:02	3	7	43%
Rv3874	EISTNIRQAGVQYSR	DQB1*03:02	3	8	38%
Rv3874	EISTNIRQAGVQYSR	DPB1*04:01	3	8	38%
Rv3615c	VDLAKSLRIAAKIYS	DRB3*02:02	3	8	38%
Rv3874	ISTNIRQAGVQYSRA	DQB1*03:02	3	9	33%
Rv3874	EISTNIRQAGVQYSR	DRB4*01:01	3	10	30%
Rv3874	ISTNIRQAGVQYSRA	DRB1*04:01	3	10	30%
		**Average**	**4**	**6**	**64%**

^a^Epitope is found in multiple antigens. Only one antigen is indicated.

It has been reported that even very dominant epitopes are typically not recognized in 100% of individuals expressing the restricting HLA molecule, suggesting incomplete penetrance. For example, in the case of the well-known HLA class I A*02:01-restricted Influenza MP_58-66_ or Hepatitis B Virus C_18-27_ epitopes, only 60–70% of individuals expressing A*02:01 recognized these epitopes in the context of natural infection [47–49]. We defined penetrance for each of the 37 most prominent epitopes restricted by a given HLA as the proportion of individuals who expressed the restricting HLA allele and who responded to the particular epitope. The average penetrance was 64% (range 30 to 100%) for all tested epitopes (Table 4), suggesting incomplete penetrance for most Mtb epitopes and underlying the complexity of responses at the population level in the context of natural infections.

### Correlation between frequency of HLA expression and HLA restriction

Our data allows estimation of the relative contributions of the four different HLA class II loci in terms of restricting the global response. The DRB3/4/5 locus restricted 32% of the total restriction events, followed by DRB1 (31%), DQB1 (27%) and DPB1 (10%). These data suggest that focusing on DRB1 and DRB3/4/5 only, a common strategy in studies of CD4 T cell responses, would allow coverage of approximately two thirds of the total HLA class II restricted response.

The data further allowed us to assess whether the population frequency of a given HLA allele was associated with the frequency of identified epitopes restricted by that allele (Table 5). Given that a frequent allele will be present in more of the donors we tested, we assumed that there would be a positive correlation. Indeed, the overall HLA phenotypic frequency strongly correlated (Spearman r = 0.70, p<0.0001) with the relative proportion of total restrictions identified for the corresponding alleles (S2 Fig). However, some exceptions were noted. For example, DPB1*01:01 was expressed by 31.7% of the donor population, but restricted only 3.7% of the identified T cell epitopes (Table 5). Thus, the correlation between population frequency and recognition frequency is not absolute.

**Table 5:** HLA genotype and phenotype frequencies, and proportions of identified epitope restrictions.

HLA allele	Genotype frequency (%)	Phenotype frequency (%)	Proportion of identified epitope restrictions (%)
DPB1*01:01	16.7	31.7	3.7
DPB1*02:01	8.7	17.5	0.6
DPB1*04:01	16.7	27.0	2.1
DPB1*04:02	6.3	12.7	4.0
DQB1*02:01	11.1	19.0	3.3
DQB1*03:01	8.7	15.9	2.7
DQB1*03:02	14.3	25.4	3.9
DQB1*04:02	4.0	6.3	1.3
DQB1*05:01	9.5	15.9	3.5
DQB1*06:02	19.0	28.6	12.3
DRB1*01:01	4.0	7.9	0.4
DRB1*03:01	11.1	22.2	5.8
DRB1*03:02	6.3	11.1	0.6
DRB1*04:01	11.1	22.2	3.7
DRB1*04:04	4.0	7.9	4.6
DRB1*04:05	4.0	7.9	1.7
DRB1*07:01	9.5	17.5	4.8
DRB1*11:01	5.6	11.1	1.7
DRB1*11:02	1.6	3.2	0.2
DRB1*12:01	4.8	9.5	1.2
DRB1*13:01	7.1	12.7	1.7
DRB1*13:02	2.4	4.8	0.4
DRB1*13:03	2.4	4.8	0.6
DRB1*15:01	4.0	7.9	3.3
DRB3*01:01	15.1	27.0	5.2
DRB3*02:02	28.6	49.2	13.7
DRB3*03:01	8.7	15.9	2.3
DRB4*01:01	15.9	28.6	2.9
DRB5*01:01	9.5	19.0	7.9

### Epitope pools as an alternative approach to characterize global Mtb-specific T cell responses

**Fig 6:**
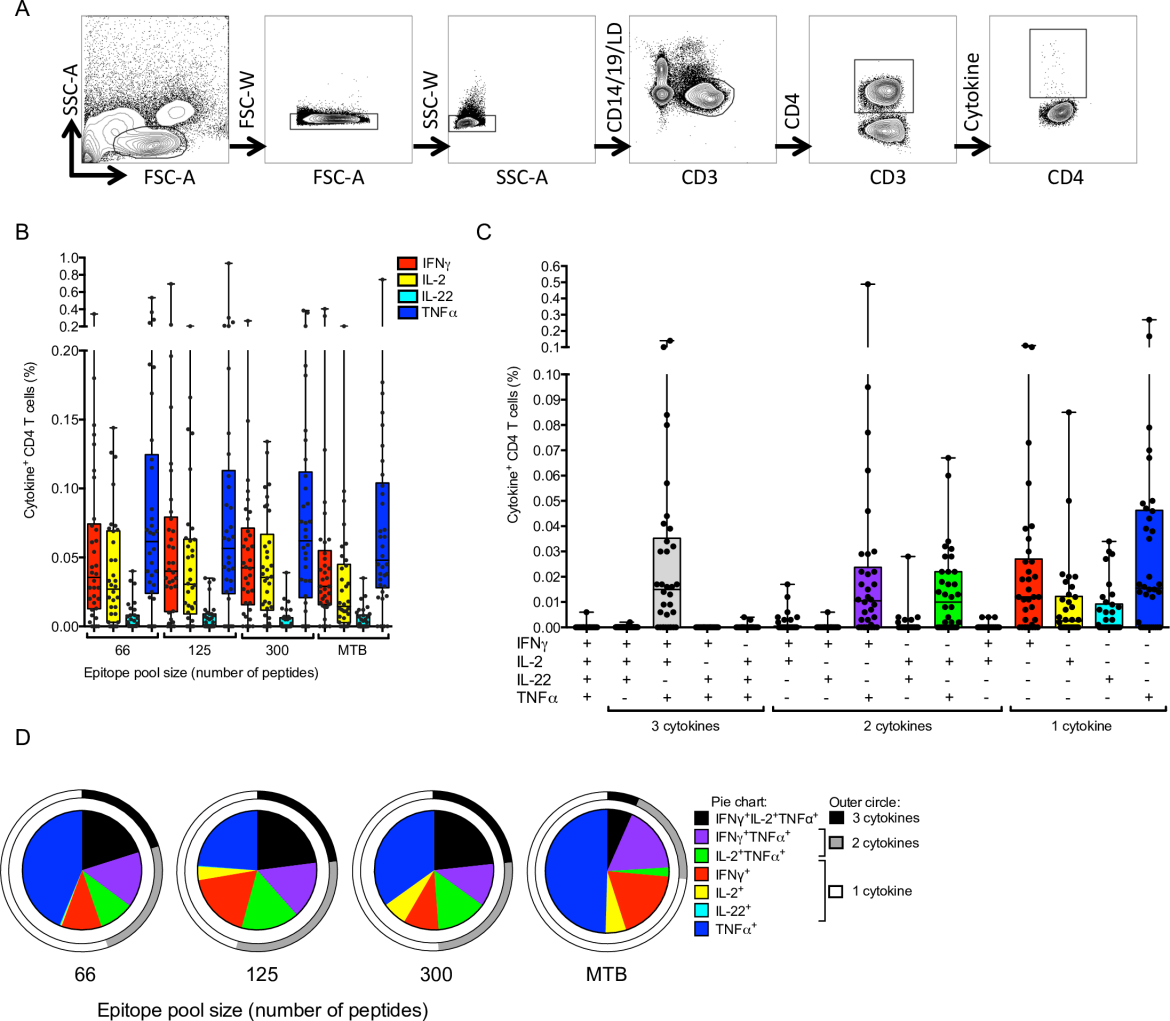
Characterization of CD4 T cell responses using epitope pools. (A) Gating strategy for ICS assay. SSC-A; Side-scatter area, FSC-A; Forward-scatter area, SSC-W; Side-scatter width, FSC-W; Forward-scatter width, LD; Live/Dead discrimination. (B) Percentage cytokine detected from CD3^+^CD4^+^ T cells in response to the pool of 66, 125 and 300 epitopes, as well as heat killed H37Rv Mtb lysate. Each dot represents one donor (n = 34) median ± interquartile range is indicated. (C) Percentage Epitope pool-specific (125 epitopes) IFNγ, TNFα, IL-2 and IL-22 production by CD3^+^CD4^+^ T cells expressing each of the fifteen possible combinations. Each dot represents one donor (n = 34) median ± interquartile range is indicated. (D) The fraction of the total cytokine response against each stimuli expressing each combination of cytokines (pie chart) and all 4, 3, 2 or 1 cytokine (outer circle).

We recently reported that sequential lyophilization allows generation and testing of peptide pools encompassing large numbers of epitopes [15,50]. As described above, we generated pools of epitopes corresponding either to the total 125 reactive epitopes or to the 66 most dominant epitopes (S5 Table). In addition, we also generated a comprehensive pool (S5 Table), that included 300 epitopes detected in the present and the previously completed genome-wide screening studies [10]. Stimulation of PBMC with these three epitope pools, as well as heat-killed Mtb lysate, was tested in an ICS assay on 34 selected donors. The gating strategy is illustrated in Fig 6A. Frequencies of cytokine-expressing CD4 T cells in response to any of the three individual epitope pools were equivalent to the Mtb lysate (Fig 6B).

The results presented above were generated by ELISPOT assays, which detect responses by both CD4 and CD8 T cells. We therefore investigated whether responses were mediated by CD4 and/or CD8 T cells. We compared the reactivity to the three epitope pools and Mtb lysate to a pool of class I and II restricted epitopes derived from Epstein-Barr Virus (EBV) and Cytomegalovirus (CMV) as control epitopes. The reactivity seen in CD4^+^ T cells was equivalent irrespective of antigen, whereas significantly higher reactivity was observed to the EBV/CMV epitope pool in CD8^+^ T cells (S3 Fig). The ICS assay detected some Mtb-specific reactivity in CD8 T cells, suggesting that responses to these epitope pools are mediated by both CD4 and CD8 T cells.

**Fig 7:**
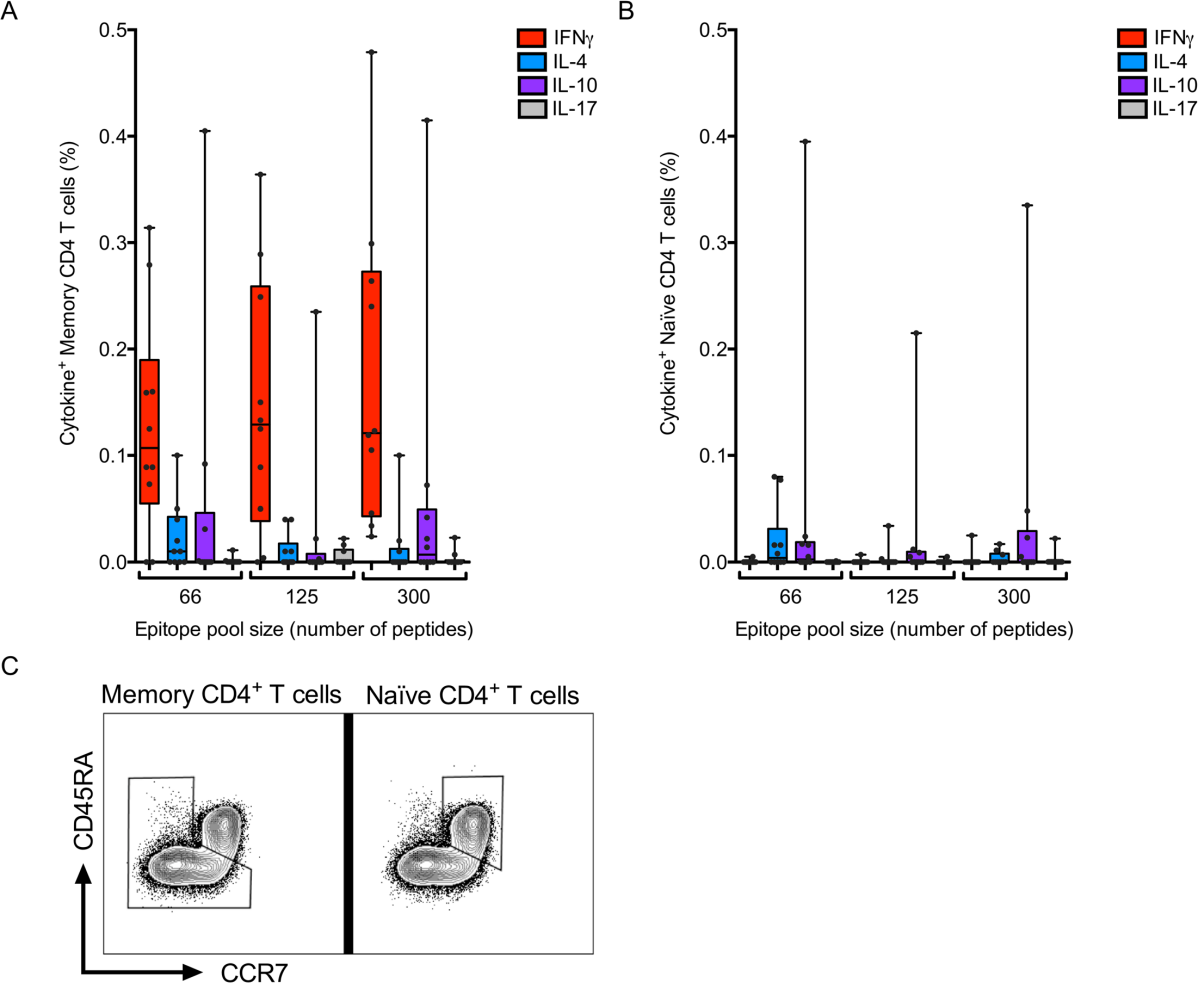
Epitope pool responses are highly polarized towards IFNγ production. Frequencies of cytokine-expressing CD3^+^CD4^+^ memory T cells (A) or CD3^+^CD4^+^ naïve T cells (B) in response to the pool of 66, 125 and 300 epitopes. Each dot represents one donor, median ± interquartile range is indicated. (C) Gating strategy for memory versus naïve CD4 T cells. Plots are gated on total CD3^+^CD4^+^ T cells.

Additionally, we investigated the production of IL-4, IL-10 and IL-17, as well as CD45RA and CCR7, to further characterize the functions and phenotypes of the CD4 T cell response to the defined peptide pools (Fig 7). As described above for ELISPOT assays, we observed no IL-4, IL-10 or IL-17 expression in response to the peptide pools (Fig 7). Furthermore, the response was exclusively mediated by memory CD4^+^ T cells and not naïve (CD45RA^+^CCR7^+^) T cells (Fig 7).

The majority of cytokine-positive CD4^+^ T cells expressed TNFα or IFNγ alone, or co-expressed IFNγ^+^TNFα^+^IL-2^+^, IFNγ^+^TNFα^+^ or TNFα^+^IL-2^+^ (Fig 6C and 6D). As expected, all peptide pools elicited IFNγ-production, however, there was heterogeneity amongst responding individual cells and a proportion of CD4^+^ T cells did not produce IFNγ. Consistent with the finding that the pool of 66 epitopes encompassed most (80%) of the total reactivity, frequencies of cytokine-expressing CD4 T cells detected with this pool were not markedly different to those detected with the larger peptide pools (Fig 6D). Similarly, no differences in cytokine co-expression patterns were observed for the different peptide pools (Fig 6D). Significantly higher proportions of CD4 T cells responding to the peptide pools co-expressed IFNγ, TNFα and IL-2 (median 48.2, IQR 31.5–78.2 for single cytokine producers), compared to Mtb-lysate reactive cells (median 63.1, IQR 44.7–87.6; p<0.01; Two-tailed Mann-Whitney), which were predominantly single cytokine producers (Fig 6D).

**Fig 8:**
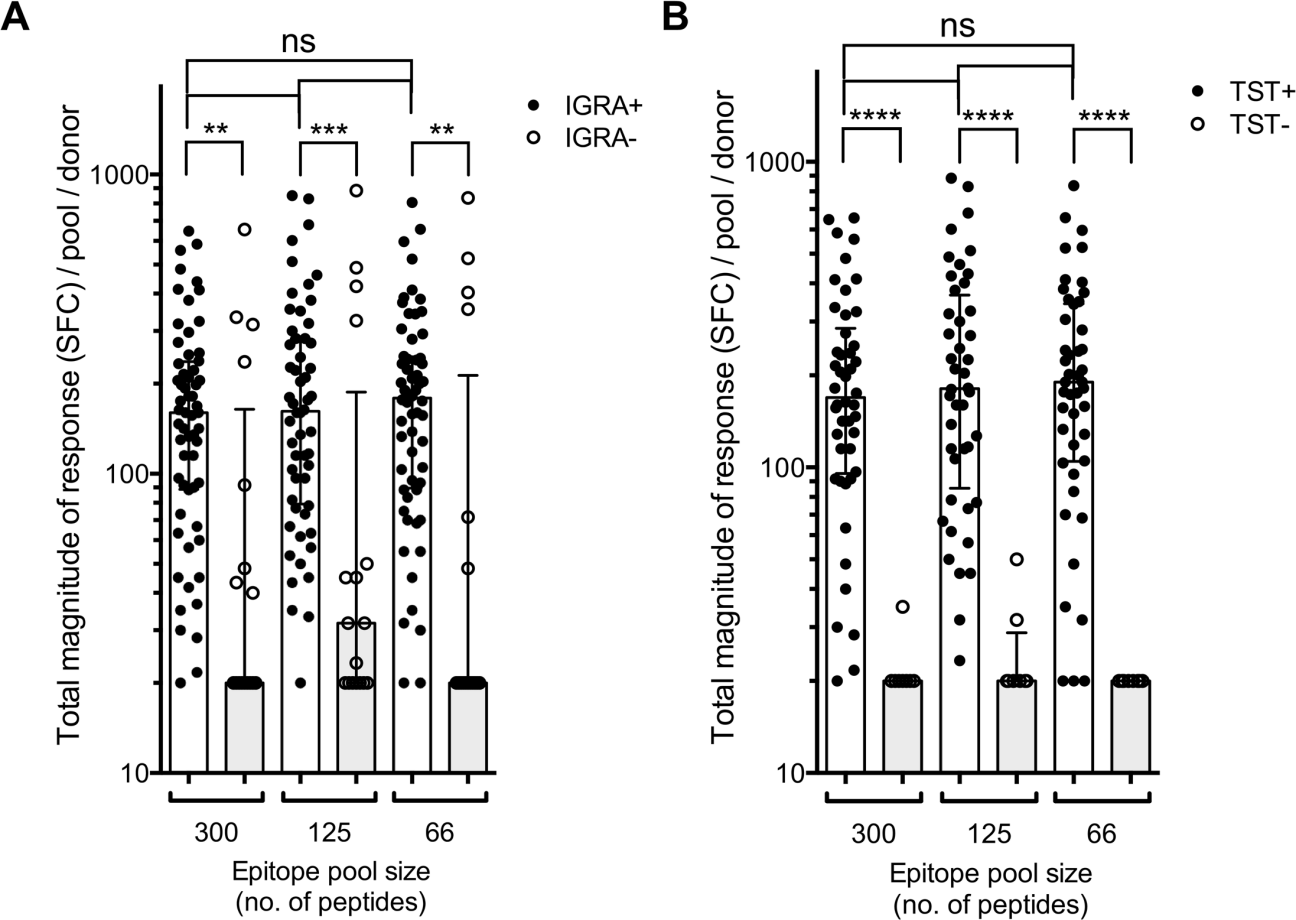
General applicability of peptide pools to detect responses in other cohorts. (A) Magnitude of epitope pool (megapool) responses in a validation cohort of individuals with LTBI, and an IGRA-negative control cohort. Each dot represents one donor (n = 60, IGRA+, black dots and n = 17, IGRA-, open circles) median ± interquartile range is indicated. Two-tailed Mann-Whitney test, ns; no significant difference, **, p<0.01, ***, p<0.001. (B) Magnitude of megapool responses in individuals in the validation and negative control groups stratified by TST status. Each dot represents one donor (n = 46, TST+, black dots and n = 8, TST-, open circles) and median ± interquartile range is indicated. Two-tailed Mann-Whitney test, ns; no significant difference, ****, p<0.0001.

Finally, to demonstrate the general applicability of these peptide pools for measuring MTB-specific CD4 T cell responses, we tested them in two independent donor cohorts, encompassing 60 IGRA-positive and 17 IGRA-negative adolescent donors from the same region of South Africa. Response reactivity and total magnitude of responses to the 66, 125 and 300 peptide pools detected in PBMC from the IGRA-positive adolescent cohort (Fig 8A) were generally high. Only very few IGRA-positive donors (<4% for all three pools) did not respond at all to the epitope pools, suggesting that these epitope sets provide broad response coverage in donors with LTBI. Furthermore, very few IGRA-negative donors from the same region and population had T cell responses to these peptide pools and those with reactivity typically had low magnitudes of responses (Fig 8A). To investigate potential bias introduced by classifying individuals based on IGRA results we stratified individuals with available TST results from the validation and control groups by their TST status. At an induration cut-off of 5mm, 46 were TST-positive and 8 were TST-negative (Table 1). As expected, responses detected from the TST-positive donors were generally high (Fig 8B). In contrast, very few TST-negative donors responded to these peptide pools, and those with responses had very low responses (Fig 8B). We also found significantly lower frequencies of IFNγ-expressing CD4 T cells in response to the peptide pools in the IGRA-negative individuals by ICS assay (S4 Fig). Frequencies of IL-2 and TNFα-expressing CD4 T cells also appeared lower in these IGRA-negative individuals compared to IGRA positive individuals. However, these differences were not striking and in some instances not significant (S4 Fig). Our data thus demonstrate that these peptide pools can be used to detect MTB-specific T cell responses by both ELISPOT and ICS assays.

## Discussion

We report the first quantitative analysis on a population level of the complexity of pathogen-specific human HLA class II-restricted CD4 T cell responses. The results are relevant both for our understanding of human responses in a natural infection setting and in the context of immune profiling strategies.

Here we estimated the complexity of Mtb-specific human T cell responses. We have defined the epitopes contained in eleven different antigens by synthetizing and testing overlapping peptides spanning the entire sequence of the antigen. We found that previously defined epitopes, based on HLA class II allele binding predictions account for about 50% of the total magnitude of response, thus providing a rough estimate of the success of genome-wide epitope mapping consistent with previously estimates [42]. By combining epitopes from the overlapping peptides spanning the eleven Mtb antigens with the predicted/previously defined epitopes we estimate that about 50–75% of the total magnitude of response can be accounted for in a population sample comprising 63 IGRA-positive donors. Therefore, we propose that additional epitopes that account for the remaining 25% of the total magnitude are yet to be discovered.

This study provided a glimpse onto the staggering complexity of population level responses in the context of a natural infection. Using a recently described comprehensive panel of single HLA class II transfected cell lines [44], we detected 778 different epitope/HLA combinations being recognized in the donor cohort. This is likely to be a gross underestimate of the actual complexity, and does not take into account CD8 T cell responses restricted by HLA class I. The epitopes we studied represent a fraction (50–75%) of the reactivity associated with latent Mtb infection. Further, restrictions could be determined only for 36% of all epitopes/donor combinations due to limitations in the number of cells available, while restrictions could be determined only for about 66% of all HLA/epitopes due to limited HLA-transfected cells available for study. Thus we estimate that the actual complexity might be 4 to 8-fold higher. We hypothesize that similar complexity, defined by highly heterogeneous epitope recognition and HLA class II restriction, is typical for the T cell response in human populations to complex pathogens that comprise multiple antigens. The implications of our findings for studies of immunopathogenesis of Mtb infection, which often focus on responses to ESAT-6/CFP10 and/or PPD, are not definitive. However, the finding that non-IGRA antigens comprise the majority of the human response to peptide antigens from Mtb suggests that future studies of immune responses should include a broader set of antigens than those in the IGRAs.

This study also allowed quantitating the relative contributions of DRB1, DRB3/4/5, DP and DQ loci to restriction of functional T cell responses. We found that all four HLA class II loci contribute to restrict responses, even though the contribution of DP appeared less prominent. This parallels previous estimates of locus contribution to CD4 T cell responses in allergy and Mtb, which suggest predominant restriction by the DR loci, with lesser, but appreciable contributions by DQ and DP [9,10,35,42,51].

Our study provided a very detailed quantitative account of complexity of human T cell responses to Mtb infection in a natural setting. The majority of epitopes were found to be highly promiscuous, as suggested by previous reports published by us and others [10,35,52–55]. It should be noted that the majority of these previously reported epitopes were predicted for promiscuous HLA binding. Promiscuity can be explained by extensive overlap in the peptide binding repertoires between different HLA alleles [56–58], and is a significant contributor to expanding complexity of human T cell responses.

We further showed that the penetrance of the vast majority of response restricting HLA alleles was highly incomplete. Hence, the fact that a given donor expresses an allele that is known to restrict a response to a given epitope does not predict definite detection of a response. While the molecular basis of this phenomenon is unclear it has been proposed that expression of other HLA alleles, or development of other, more immunodominant responses may be responsible for varying shifts in immunodominance from individual to individual [19].

Our results are also relevant in terms of the different strategies currently being developed for immunoprofiling of immune responses in the context of vaccination or natural infection. Specifically, the dataset generated allowed us to calculate a quantitative estimate of how many different HLA class II tetramers would be required to cover Mtb-specific responses in this exemplary population. In the present study we determined HLA restrictions for 278 of the positive epitope/donor combinations out of a total of 778 (36%), which accounted for 47% of the total reactivity. Because the same epitope can be restricted by more than one HLA allele in the donor population, a total of 380 HLA/epitope combinations were possible. The best response coverage would thus require construction of 380 unique HLA class II tetramers, reaching 30% coverage of the total magnitude of response (63% coverage by HLA transfected cell lines × 47% of total magnitude covered by determined restrictions). Thus, if several hundred different HLA class II tetramers were made, these would cover only about 30% of the response and 70% of the response would remain undetected. Practical considerations would further compound this issue. Tetramer production has not been validated for all HLA class II allelic variants, with certain alleles being more technically challenging than others, and not all epitopes readily yield functional tetramers with some epitopes associated with poor yields and others with high non-specific staining.

Despite significant advances in tetramer multiplexing and advances in the development of HLA class II tetramers [59–62], a tetramer based approach may be better reserved to characterize examples of responses restricted by a particular HLA and a specific peptide, and thereby possibly unsuited to characterize responses in a whole population or in a large collection of antigens. A potential issue in characterizing responses using only one or few “prototypic” epitopes is that the available data suggests that the T cell phenotypes associated with different epitopes might be heterogenous and highly complex [63]. In addition, T cells recognizing “decoy” antigens, or T cells crossreactive with other pathogens have been described [64,65], raising the issue that characterization of isolated “representative” epitopes might not be representative of the full breadth of responses.

Pooling of many peptides into “megapools” of epitopes, by sequential lyophilization for antigen stimulation assays, such as ICS, may be a more practical approach for response characterization, especially if only small amounts of cells are available. Although our approach was intended for discovery and characterization of CD4 T cell epitopes, we found that CD8 responses can be detected with these peptide pools. This is not unexpected since CD8 T cell epitopes are contained within the same sequences as the CD4 T cell epitopes. Our results thus demonstrate that Mtb megapools encompassing promiscuously recognized peptides can be used effectively to phenotype Mtb-specific T cell responses. We expect these megapools to have wide applicability irrespective of ethnicity and geographical location since previous studies have shown recognition of many of the peptides in diverse human populations and even non-human primates [10,66,67]. Recent data utilizing a similar approach in the context of dengue virus-specific responses in endemic areas, EBV and CMV-specific, as well as Pertussis-specific T cell responses suggest that this approach will have general applicability (manuscript in preparation).

IGRA-negative individuals had no responses or much lower T cell response frequencies to the Mtb megapools than IGRA-positive individuals. This result was not surprising since the IGRA-negative donors were recruited from a setting where infant BCG vaccination is routine, while exposure to environmental mycobacteria is also likely. The low level reactivity of IGRA-negative individuals most likely reflects immunological sensitization by BCG and/or environmental mycobacteria [65]. Interestingly, TST-negative individuals had essentially no response to the Mtb megapools, indicating that the T cell response we can detect with this broad range of antigens reflects mycobacterial sensitization detected by TST. In vitro immunological assays that measure responses to the Mtb megapools may serve as a useful alternative to this test. Further studies will evaluate this in more detail.

The results also have direct implications for our understanding of Mtb-specific T cell responses. Our study defined a large set of epitopes with defined HLA restrictions from broad range of Mtb antigens and report the response breadth and population coverage that can be attained with these epitopes. We propose that these well-defined epitopes are a valuable resource for immunological characterization of Mtb-specific T cell responses, either as individual or pooled peptides, or for construction of HLA class II tetramer reagents. Ongoing studies are investigating the chemokine receptor expression of the responding T cells in this cohort, which will be informative in further characterizing the Mtb-specific T cell responses.

A recent study underlined that bi-allelic RORC loss-of-function mutations resulted in the absence of IL-17-producing T cells and defective IFNγ production by circulating γδ T cells and CD4^+^CCR6^+^CXCR3^+^ αβ T cells, and was associated with mycobacteriosis [26]. Our parallel measurement of IL-5, IL-17, IL-10 and IFNγ-expressing cells did not discover novel Mtb-specific epitope-reactive CD4 T cells that did not produce IFNγ. In fact, as expected for IGRA-positive individuals, the Mtb-specific response was highly polarized towards IFNγ production. Our data also confirmed the previous finding that the CXCR3^+^CCR6^+^ Th subset of Mtb-specific CD4 T cells produced IFNγ, IL-2 and/or TNFα, and not IL-17 in response to Mtb epitopes [8].

Our analysis of responses to TB vaccine and IGRA antigens show that different antigens are recognized with markedly different prevalence and magnitude of response. An association between immunogenicity and biological function of the antigenic target was apparent. Antigens in the cell wall and cell processes category were the most immunogenic, followed by PE/PPE proteins. These data are consistent with our previous report [10], which suggested that certain functional and structural features dictate reactivity of TB antigens in Mtb-infected persons. In terms of implications for vaccine selection, and in agreement with a recent analysis of reactivity to 59 different TB antigens in geographical locations ranging from North, Central and South America, Europe, Africa and Asia [66], the data demonstrates that certain antigens are associated with poor immunogenicity in the context of natural TB infection. The implications for incorporation of poorly immunogenic proteins into TB vaccines are not clear. It is possible that vaccination-induced T cell responses to such antigens may constitute so-called “unnatural” immunity, which is distinct from that primed by natural Mtb infection. It has been hypothesized that vaccine-induced protection over and above that provided by natural immunity may be achieved by induction of “unnatural” immune responses [68]. On the other hand, it could also be that the “unnatural” antigens are simply not visible to the immune system during infection and are unprotective. These questions will be answered after completion of the ongoing vaccine trials that encompass these antigens.

In conclusion, this study provides an in-depth characterization of Mtb antigens and epitopes recognized, and the response-restricting HLA class II molecules, in a cohort of Mtb-infected donors from the TB-endemic Western Cape region of South Africa. The results provide a case study, comparing different strategies to globally characterize immune reactivity against an infectious agent in a human population.

## Materials and Methods

### Ethics statement

Research conducted for this study was performed in accordance with approvals from the Human Research Ethics Committee of the University of Cape Town. All participants provided written informed consent prior to participation in the study. In the case of adolescents, they provided written informed assent and written informed consent was also provided by a parent or legal guardian.

### Study subjects

For T cell response screening we recruited 63 healthy adults with latent Mtb infection from the Worcester region of the Western Cape Province of South Africa. LTBI was confirmed by a positive IGRA (QuantiFERON-TB Gold In-Tube, Cellestis) (Table 1), which is the most robust assay for identification of individuals with LTBI in a BCG vaccinated population [69]. We previously reported good agreement between tuberculin skin test (TST) and IGRA in this study population, and no significant differences in clinical, epidemiological or immunological attributes between IGRA positive and TST positive persons have been noted [70]. We therefore did not consider TST when defining LTBI. All participants tested negative for HIV infection. All participants provided written informed consent for participation in the study. Venous blood was collected in heparin-containing 450 ml blood bags.

For the “validation cohort” we retrieved cryopreserved PBMC from 60 IGRA-positive, healthy adolescents previously enrolled into the adolescent cohort study (South African Tuberculosis Vaccine Initiative, University of Cape Town) [71], also performed in the Worcester region of South Africa (Table 1). Adolescents provided written informed assent and written informed consent was also provided by a parent or legal guardian. HIV testing of these healthy adolescents was not done. However, since the prevalence of HIV seropositivity in TB cases in the adolescent cohort study was less than 2% [72], we reasoned that the HIV prevalence amongst healthy adolescents would be negligible.

For the negative control cohort we retrieved cryopreserved PBMC from 17 IGRA-negative, healthy adolescents (Table 1) previously enrolled in the adolescent cohort study as described above (South African Tuberculosis Vaccine Initiative, University of Cape Town). Research conducted on all three cohorts above was performed with approvals from the Human Research Ethics Committee of the University of Cape Town.

### Peptides

Peptides were synthesized as crude material on a small (1 mg) scale by A and A (San Diego). Peptides representing the vaccine and IGRA antigens (Rv3874; CFP10 and Rv3875; ESAT-6) were 15-mers overlapping by 10 amino acids spanning each entire protein (Table 2). Previously described epitopes were from the frequently recognized antigens reported by Arlehamn et al. [10], as well as additional frequently recognized epitopes described in ex vivo experiments and available in the IEDB (www.iedb.org) [35,39–41,49]. S5 Table delineates the peptides in each megapool.

### PBMC isolation

Peripheral blood mononuclear cells (PBMC) were purified from whole blood either using CPT tubes (BD), for the adolescents, or layered onto Ficoll (for adults) using 50ml Leukosep tubes (Greiner) by density-gradient centrifugation, according to the manufacturer’s instructions. Cells were cryopreserved in liquid nitrogen suspended in FBS (company) containing 10% (vol/vol) DMSO. The cryopreserved cells were shipped to La Jolla Institute for Allergy and Immunology for analysis.

### ELISPOT assay

PBMC were stimulated at 2×10^5^ cells/well in triplicate with peptide pools (5 μg/ml/peptide), peptides (10 μg/ml), megapools (2 μg/ml/peptide), PHA (10μg/ml) or medium containing 0.25% DMSO (percent DMSO in the pools, as a control) in 96-well plates (Immobilion-P; Millipore) coated with anti-cytokine antibody. For single cytokine ELISPOT 5μg/ml anti-IFNγ (1-D1K; Mabtech) was used. For dual ELISPOT (IFNγ/IL-5 and IL-10/IL-17) 10μg/ml each of anti-IFNγ (1-D1K), anti-IL-5 (TRFK5), anti-IL-10 (9D7), and anti-IL-17A (MT44.6; all from Mabtech) were used. After 20h incubation at 37°C, wells were washed with PBS/0.05% Tween 20 and incubated with biotinylated anti-IFNγ (7-B6-1; single cytokine ELISPOT; Mabtech) for 2h. For dual ELISPOT, anti-IFNγ-HRP (7-B6-1-HRP), biotinylated anti-IL-5 (5A10), anti-IL-10-ALP (12G8), and biotinylated anti-IL-17 (MT504; all from Mabtech) were used. Spots were developed using Vectastain APC peroxidase (Vector Laboratories) and 3-amino-9-ethylcarbazole (Sigma-Aldrich) for single ELISPOT. For dual ELISPOT biotinylated antibodies were developed exactly like the single ELISPOT; HRP and ALP conjugated antibodies were developed by alkaline phosphatase complex and then visualized by applying the Vector Blue Alkaline Phosphatase Substrate Kit III (both from Vector Laboratories). Spots were counted by computer-assisted image analysis (KS-ELISPOT reader; Zeiss). Responses were considered positive if the net spot-forming cells (SFC) per 10^6^ PBMC were ≥20, the stimulation index ≥2, and p≤0.05 (Student’s t-test, mean of triplicate values of the response against relevant pools or peptides vs. the DMSO control). All samples had a viability >75%, as determined by trypan blue, and reactivity to PHA >400 SFC/10^6^ cells. The total magnitude of response per participant was defined as the sum of SFCs per 10^6^ PBMC detected against separate epitopes or peptide pools. The total magnitude of response across participants was defined as the sum of responses per individual.

### HLA typing

Four-digit HLA typing was performed as recently described [45]. Genomic DNA was isolated from PBMC using standard techniques (REPLI-g; Qiagen). Amplicons for HLA class I and class II genes were generated using PCR and locus-specific primers. Amplicons of the correct size were purified using Zymo DNA Clean-up Kit, according to the manufacturer’s instructions. Sequencing libraries were prepared using Nextera XT reagents (Illumina), according to manufacturer’s instructions. The libraries were purified using AMPure XP (Beckman Coulter) with a ratio of 0.5:1 beads to DNA (vol/vol). The libraries were pooled in equimolar amounts and loaded at 5.4pM on one MiSeq flowcell with 1% phiX spiked in (MiSeq Reagent Kit v3). Paired-end sequencing was performed with 300 cycles in each direction. HLA typing calls were made using HLATyphon (https://github.com/LJI-Bioinformatics/HLATyphon).

### Population coverage

For DP, DQ, DRB1 and DRB3/4/5 frequencies we considered only the beta chain frequencies, given that the DRA chain is largely monomorphic and that differences in DPA are not thought to significantly influence peptide binding [73].

### HLA restriction using single HLA transfected cell lines

Single HLA transfected RM3 (derived from human B lymphocyte cell line Raji) or DAP.3 (L cell fibroblast) were maintained as previously described [44] (all cell lines were from La Jolla Institute for Allergy and Immunology). The cell lines were harvested and viability (all >75%) was determined using Trypan Blue. Each cell line at 2x10^5^ cells/well was pulsed with 10μg/ml individual peptide for 1h at 37°C, followed by four washes in RPMI. PBMC at 2x10^5^/well were stimulated in triplicate with peptide pulsed cell line (5x10^4^ cells/well), cell line alone (as a control), peptides (10μg/ml), PHA (10μg/ml) or medium containing 0.25% DMSO (percent DMSO in the peptides, as a control) in 96-well plates (Immobilion-P; Millipore) coated with anti-IFNγ antibody as described above for single cytokine ELISPOT. Criteria for positive responses were as described for ELISPOT assays above.

### Multi-epitope peptide pools

Individual peptides were resuspended in DMSO and equal amounts of each peptide were pooled to construct peptide pools. Each peptide pool was placed in an individual lyophilizing flask (VirTis) and lyophilized on VirTis Freezemobile 35 EL for 24 hours. The semisolid product was re-suspended in water, frozen and then lyophilized again. This process was repeated until only solid product remained after lyophilization. The resulting lyophilized peptide pool was re-suspended in DMSO at a higher concentration per peptide (0.7-2mg/ml per peptide depending on number of peptides in the pool) than before lyophilization, to minimize DMSO concentrations in the assays.

### Intracellular cytokine staining

PBMC at 1x10^6^ per condition were stimulated with peptide pools (2μg/ml), or heat killed H37Rv (5x10^5^ CFU/million PBMC), together with anti-CD28 (1μg/ml) and anti-CD49d (1μg/ml) for 5h in complete RPMI medium at 37°C with 5% CO_2_. After 5h, 2.5μg/ml each of BFA and monensin was added for an additional 7h at 37°C. Unstimulated PBMCs were used to assess nonspecific/background cytokine production and PHA stimulation at 5μg/ml was used as a positive control. After a total of 12h, cells were harvested and stained for cell surface antigens CD4 (anti-CD4-APCEf780, RPA-T4, eBioscience), CD3 (anti-CD3-AF700, UCHT1, BD Pharmingen), CD8 (anti-CD8-BV650, RPA-T8, Biolegend), CD14 (anti-CD14-V500, M5E2, BD Pharmingen), CD19 (anti-CD19-V500, HIB19, BD Pharmingen), and fixable viability dye eFluor 506 (eBiosciences). After washing, cells were fixed using 4% paraformaldehyde and permeabilized using saponin buffer. Cells were stained for IFNγ (anti-IFNγ-FITC, AS.B3, eBioscience), IL-2 (anti-IL-2-PECy7, MQ1-17H12, eBioscience), TNFα (anti-TNFα-eF450, MAb11, eBioscience), and IL-22 (anti-IL-22-PerCPeFluor710, 22URTI, eBioscience) in saponin buffer containing 10% FBS. To investigate the production of IFNγ, IL-10, IL-4 and IL-17, as well as the memory phenotype, PBMC at 1x10^6^ per condition were stimulated with peptide pools (2μg/ml), together with anti-CD28 (1μg/ml) and anti-CD49d (1μg/ml) for 2h in complete RPMI medium at 37°C with 5% CO_2_. After 2h, 2.5μg/ml each of BFA and monensin was added for an additional 4h at 37°C. Unstimulated PBMCs were used to assess nonspecific/background cytokine production and PMA/Ionomycin stimulation at 1μg/ml was used as a positive control. After a total of 6h, cells were harvested and stained for cell surface antigens CD4 (anti-CD4-APCEf780, RPA-T4, eBioscience), CD3 (anti-CD3-AF700, UCHT1, BD Pharmingen), CD8 (anti-CD8-V500, RPA-T8, BD Pharmingen), CD14 (anti-CD14-V500, M5E2, BD Pharmingen), CD19 (anti-CD19-V500, HIB19, BD Pharmingen), CD45RA (anti-CD45RA-eFluor 450, HI100, eBioscience), CCR7 (anti-CCR7-PerCPCy5.5, G043H7, BioLegend) and fixable viability dye eFluor 506 (eBiosciences). After washing, cells were fixed using 4% paraformaldehyde and permeabilized using saponin buffer. Cells were stained for IFNγ (anti-IFNγ-FITC, AS.B3, eBioscience), IL-4 (anti-IL-2-BV605, MP4-25D2, BioLegend), IL-10 (anti-IL-10-APC, JES3-19F1, BioLegend), and IL-17A (anti-IL-17A-PECy7, eBio64DEC17, eBioscience) in saponin buffer containing 10% FBS. Samples were acquired on a BD LSR II flow cytometer. Frequencies of CD4^+^ or CD8^+^ T cells responding to each peptide pool were quantified by determining the total number of gated CD4^+^ or CD8^+^ and cytokine^+^ cells and background values subtracted (as determined from the medium alone control) using FlowJo X Software. Combinations of cytokine producing cells were determined using Boolean gating.

## Supporting Information

S1 Fig Reactivity to IGRA and Non-BCG antigens versus other Mtb antigens. (A) Total magnitude of epitope responses in the screening cohort divided into epitopes that map to IGRA antigens versus other Mtb antigens. Each dot represents one donor, median ± interquartile range is indicated. Two-tailed Mann-Whitney test, ****, p<0.0001. (B) Total magnitude of epitope responses in the screening cohort divided into epitopes that map to antigens missing from BCG versus other Mtb/BCG shared antigens. Each dot represents one donor, median ± interquartile range is indicated. Two-tailed Mann-Whitney test, *, p<0.05.
(EPS)

S2 Fig HLA allele frequencies correlates with number of identified epitope restrictions. Correlation of HLA allele phenotype frequency (y-axis) versus percentage restrictions identified for each HLA allele (x-axis). Line indicates linear regression. Correlation is indicated by Spearman r and associated two-tailed p-value.
(EPS)

S3 Fig Epitope pool responses are mediated by CD4^+^ T cells. Percentage cytokine detected from CD3^+^CD4^+^ T cells (A) or CD3^+^CD8^+^ T cells (B) in response to the pool of 66, 125 and 300 epitopes, as well as heat killed H37Rv Mtb lysate and a pool of EBV/CMV epitopes. Each dot represents one donor, median ± interquartile range is indicated. One-tailed Mann-Whitney test, **, p<0.01, ****, p<0.0001.
(EPS)

S4 Fig MTB-specificity of the peptide pools. Percentage cytokine detected from CD3^+^CD4^+^ T cells in response to the pool of 66, 125 and 300 epitopes, as well as heat killed H37Rv Mtb lysate. Each dot represents one donor (n = 34, IGRA+, black dots and n = 17 for 66 and 300, n = 16 for 125 and MTB, IGRA-, grey dots) median ± interquartile range is indicated. One-tailed Mann-Whitney test, ns; no significant difference, *, p<0.05, **, p<0.01, ***, p<0.001, ****, p<0.0001. (A) IFNγ, (B) IL-2, and (C) TNFα.
(EPS)

S1 Table The most commonly recognized 37 epitopes defined in TB Vaccine and IGRA antigens. (DOCX)

S2 Table The most commonly recognized 38 epitopes defined from previously described epitopes. (DOCX)

S3 Table HLA type of adult donor cohort. (XLSX)

S4 Table HLA restriction and penetrance for Mtb epitopes. (XLSX)

S5 Table Peptides in each megapool. (XLSX)
