# Supplementary material for: A Quantitative Analysis of Complexity of Human Pathogen-Specific CD4 T Cell Responses in Healthy M. tuberculosis Infected South Africans
Source: PLoS Pathog. 2016 Jul 13;12(7):e1005760. doi: 10.1371/journal.ppat.1005760 (PMC4943605; doi:10.1371/journal.ppat.1005760)
Supplement: S1 Table — (DOCX) [file ppat.1005760.s005.docx]

**Table S1. The most commonly recognized 37 epitopes defined in TB Vaccine and IGRA antigens**

| **Category** | **Antigen** | **Epitope sequence** | **Responding donors, n** | **Total magnitude of response (SFC)** |
| --- | --- | --- | --- | --- |
| Cell wall and cell processes | Rv0288 | MSQIMYNYPAMLGHA | 12 | 2461 |
|  |  | SAWQGDTGITYQAWQ | 10 | 1215 |
|  |  | HEANTMAMMARDTAE | 10 | 920 |
|  |  | YAGTLQSLGAEIAVE | 5 | 958 |
|  |  | VRAYHAMSSTHEANT | 5 | 868 |
|  |  | YQAWQAQWNQAMEDL | 2 | 63 |
|  | Rv3619c | DAHGAMIRAQAGSLE | 13 | 1773 |
|  |  | MTINYQFGDVDAHGA | 3 | 471 |
|  |  | FQVIYEQANAHGQKV | 3 | 221 |
|  |  | HGQKVQAAGNNMAQT | 3 | 552 |
|  | Rv3620c | MTSRFMTDPHAMRDM | 4 | 633 |
|  |  | VEDEARRMWASAQNI | 2 | 268 |
|  |  | ATSLDTMTQMNQAFR | 2 | 65 |
|  |  | NIVNMLHGVRDGLVR | 2 | 307 |
|  | Rv3874 | EISTNIRQAGVQYSR | 19 | 3753 |
|  |  | AQAAVVRFQEAANKQ | 12 | 1527 |
|  |  | GQWRGAAGTAAQAAV | 5 | 167 |
|  |  | ADEEQQQALSSQMGF | 4 | 280 |
|  |  | KTQIDQVESTAGSLQ | 3 | 466 |
|  |  | GNFERISGDLKTQID | 2 | 243 |
|  | Rv3875 | MTEQQWNFAGIEAAA | 8 | 356 |
|  |  | NLARTISEAGQAMAS | 8 | 643 |
|  |  | SAIQGNVTSIHSLLD | 4 | 295 |
|  |  | EGKQSLTKLAAAWGG | 4 | 165 |
|  |  | YQGVQQKWDATATEL | 3 | 777 |
| Intermediary metabolism and respiration | Rv0125 | VAQVGPQVVNINTKL | 3 | 387 |
| Lipid metabolism | Rv1886c | AGSLSALLDPSQGMG | 3 | 722 |
|  | Rv3804c | VPSPSMGRDIKVQFQ | 2 | 97 |
| PE/PPE | Rv1196 | ALPPEINSARMYAGP | 6 | 525 |
|  |  | TPAIAVNEAEYGEMW | 6 | 1798 |
|  |  | VPPPVIAENRAELMI | 4 | 533 |
|  |  | LIATNLLGQNTPAIA | 4 | 341 |
|  |  | VAAAQMWDSVASDLF | 3 | 125 |
|  |  | MVAAASPYVAWMSVT | 3 | 385 |
|  |  | YGEMWAQDAAAMFGY | 3 | 183 |
|  |  | AAVEEASDTAAANQL | 2 | 66 |
|  | Rv2608 | LPPEVNSARIFAGAG | 2 | 400 |
