# Supplementary material for: A Quantitative Analysis of Complexity of Human Pathogen-Specific CD4 T Cell Responses in Healthy M. tuberculosis Infected South Africans
Source: PLoS Pathog. 2016 Jul 13;12(7):e1005760. doi: 10.1371/journal.ppat.1005760 (PMC4943605; doi:10.1371/journal.ppat.1005760)
Supplement: S2 Table — (DOCX) [file ppat.1005760.s006.docx]

**Table S2. The most commonly recognized 38 epitopes defined from previously described epitopes.**

| **Antigen^a^** | **Epitope sequence** | **Responding donors, n** | **Total magnitude of response (SFC)** | **Reactivity in both sets^b^** |
| --- | --- | --- | --- | --- |
| Rv0124 | AQIYQAVSAQAAAIH^a^ | 2 | 73 |  |
| Rv0129c | PSPSMGRDIKVQFQS^a^ | 3 | 140 |  |
| Rv0256c | AVLVATNFFGINTIP^a^ | 6 | 455 |  |
| Rv0280 | GINTIPIAINEAEYV^a^ | 7 | 1346 |  |
| Rv0288 | AAFQGAHARFVAAAA^a^ | 12 | 1355 |  |
|  | AAGTYVAADAAAAST^a^ | 4 | 315 |  |
|  | MSQIMYNYPAMMAHA^a^ | 10 | 1495 | Yes |
|  | EDLVRAYHAMSSTHE^a^ | 6 | 903 | Yes |
|  | LQSLGAEIAVEQAAL^a^ | 2 | 265 | Yes |
| Rv1047 | AGWLAFFRDLVARGL^a^ | 2 | 52 |  |
|  | ASIIRLVGAVLAEQH^a^ | 2 | 155 |  |
| Rv1172c | ALSRVQSMFLGTGGS | 2 | 142 |  |
|  | MSFVTTQPEALAAAA^a^ | 6 | 640 |  |
| Rv1195 | MHVSFVMAYPEMLAA | 4 | 637 |  |
|  | SSYAATEVANAAAGQ | 3 | 372 |  |
| Rv1196 | LGGLWTAVSPHLSPL | 2 | 210 |  |
|  | RSPISNMVSMANNHM | 2 | 108 |  |
| Rv1705c | FFGQNTAAIAATEAQ^a^ | 4 | 551 |  |
| Rv1886c | AGCQTYKWETFLTSE^a^ | 3 | 76 |  |
| Rv1908c | WTNTPTKWDNSFLEI | 2 | 165 |  |
| Rv1926c | VALAAYPITGKLGSE | 2 | 133 |  |
| Rv2031c | AYGSFVRTVSLPVGA | 5 | 273 |  |
| Rv2770c | LPPEVNSARMYGGAG | 6 | 600 |  |
| Rv3024c | AEKFKEDVINDFVSS | 2 | 100 |  |
| Rv3025c | ILPIAEMSVVAMEFG | 5 | 287 |  |
| Rv3136 | LLGQNTAAIAAIEAQ | 4 | 520 |  |
| Rv3330 | LENDNQLLYNYPGAL | 6 | 306 |  |
|  | MAFLRSVSCLAAAVF | 2 | 75 |  |
| Rv3615c | LRIAAKIYSEADEAW | 14 | 1860 |  |
| Rv3873 | APLAQEREEDDEDDW | 4 | 210 |  |
| Rv3874 | QAAVVRFQEAANKQK | 18 | 2481 | Yes |
|  | ISTNIRQAGVQYSRA | 18 | 4035 | Yes |
|  | QGQWRGAAGTAAQAA | 3 | 95 | Yes |
| Rv3875 | EQQWNFAGIEAAASA | 19 | 1426 | Yes |
|  | IQGNVTSIHSLLDEG | 8 | 942 | Yes |
|  | ARTISEAGQAMASTE | 8 | 612 | Yes |
|  | ELNNALQNLARTISE | 4 | 932 |  |
| Rv3876 | RQSGATIADVLAEKE | 2 | 87 |  |

^a^Epitope is found in multiple antigens. Only the antigen with lowest Rv number is indicated.

^b^Reactivity to an epitope overlapping by at least 11 amino acids were also detected in the TB Vaccine and IGRA antigens.
